# Supplementary material for: Effects of Aging and Methionine Restriction on Rat Kidney Metabolome
Source: Metabolites. 2019 Nov 14;9(11):280. doi: 10.3390/metabo9110280 (PMC6918429; doi:10.3390/metabo9110280)
Supplement: Supplementary file 1 [file metabolites-09-00280-s001.pdf]

Table S1. Details of the unidentified lipid and metabolite species in rat renal cortex which displayed statistically significant differences between the *Adult* and *Aged* groups. Results were obtained by a non-parametric t-test of the data obtained using an untargeted lipidomic and metabolomic profiling method based on LC-MS with ESI(+).

Method	Compound	m/z	RT	Log FC	p value	*Aged* vs *Adult*
Lipidomic	108.088@0.83	109.088	0.83	8.56	2.40E-02	up
Lipidomic	126.0983@0.83	127.0983	0.83	-7.48	3.05E-02	down
Lipidomic	271.1728@0.80	272.1728	0.8	10.15	2.64E-03	up
Lipidomic	343.2165@0.79	344.2165	0.79	8.65	2.84E-02	up
Lipidomic	395.3869@5.26	396.3869	5.26	9.06	3.37E-02	up
Lipidomic	431.23@0.80	432.23	0.8	-6.81	4.79E-02	down
Lipidomic	626.5172@0.84	627.5172	0.84	-8.87	9.29E-03	down
Lipidomic	686.1922@8.07	687.1922	8.07	-7.61	2.61E-02	down
Lipidomic	701.2069@5.12	702.2069	5.12	-10.06	4.35E-03	down
Lipidomic	702.5582@0.88	703.5582	0.88	-7.2	3.13E-02	down
Lipidomic	716.2407@3.26	717.2407	3.26	7.43	2.97E-02	up
Lipidomic	833.4248@1.01	834.4248	1.01	10.1	4.95E-03	up
Lipidomic	914.81@9.91	915.81	9.91	-9	2.58E-02	down
Lipidomic	947.8471@10.05	948.8471	10.05	7.92	2.32E-02	up
Lipidomic	960.3313@9.21	961.3313	9.21	7.97	2.80E-02	up
Lipidomic	1091.8438@7.18	1092.8438	7.18	7.87	1.03E-02	up
Lipidomic	1111.3708@9.53	1112.3708	9.53	-7.92	2.12E-02	down
Lipidomic	1135.923@7.71	1136.923	7.71	-8.83	8.98E-03	down
Lipidomic	1218.3806@9.78	1219.3806	9.78	-7.89	2.23E-02	down
Lipidomic	1428.3749@10.01	1429.3749	10.01	7.07	2.81E-02	up
Lipidomic	1612.4502@10.18	1613.4502	10.18	-7.88	1.30E-02	down
Lipidomic	1704.4688@9.07	1705.4688	9.07	-8.02	2.87E-02	down
Metabolomic	301.291@8.57	302.291	8.57	-16.75	5.79E-04	down
Metabolomic	1233.3395@13.27	1234.3395	13.27	-9.27	1.37E-03	down
Metabolomic	809.6256@13.48	810.6256	13.48	-17.77	2.02E-03	down
Metabolomic	909.5038@14.07	910.5038	14.07	-11.56	4.13E-03	down
Metabolomic	590.4992@13.34	591.4992	13.34	-10.13	6.27E-03	down
Metabolomic	399.2837@6.21	400.2837	6.21	-11.32	7.08E-03	down
Metabolomic	734.5935@13.47	734.5935	13.47	-14.8	9.10E-03	down
Metabolomic	751.5833@13.20	752.5833	13.2	-13.71	1.13E-02	down
Metabolomic	950.5492@13.25	951.5492	13.25	-9.96	1.15E-02	down
Metabolomic	472.078@6.02	473.078	6.02	6.11	1.15E-02	up
Metabolomic	751.6068@13.20	752.6068	13.2	11.22	1.52E-02	up
Metabolomic	777.0916@10.56	778.0916	10.56	-9.35	1.59E-02	down
Metabolomic	276.1407@7.96	277.1407	7.96	6.15	1.97E-02	up
Metabolomic	753.5714@13.07	754.5714	13.07	-8.25	2.00E-02	down
Metabolomic	736.1945@11.50	737.1945	11.5	7.04	2.11E-02	up
Metabolomic	459.2482@7.44	460.2482	7.44	-7.41	2.60E-02	down
Metabolomic	381.7217@7.33	382.7217	7.33	-10.65	2.79E-02	down
Metabolomic	501.4028@0.55	502.4028	0.55	-5.58	3.22E-02	down
Metabolomic	605.3785@11.58	606.3785	11.58	-10.28	3.42E-02	down
Metabolomic	809.6344@13.48	810.6344	13.48	12.05	3.47E-02	up
Metabolomic	716.5595@12.94	716.5595	12.94	-8.83	3.69E-02	down
Metabolomic	297.2689@8.66	298.2689	8.66	-9.61	4.22E-02	down

Metabolomic	306.0749@0.46	307.0749	0.46	-9.62	4.43E-02	down
Metabolomic	157.1466@7.11	158.1466	7.11	-7.05	4.81E-02	down
Metabolomic	850.7687@14.45	851.7687	14.45	-6.98	4.82E-02	down

Table S2. Details of the unidentified renal lipid species with statistically significant differences between the *Adult* control, *Aged* control and *MetR* groups. Results were obtained by a one-way ANOVA with a post hoc Tukey's test of the data obtained using an untargeted lipidomic profiling method based on LC-MS with ESI(+).

Method	Compound	m/z	RT	p value	LogFC RMet vs Old	RMet vs Old	LogFC RMet vs Adult	RMet vs Adult
Lipidomic	431.228@0.80	432.228	0.8	2.42E-02	7.21	up	-2.04	down
Lipidomic	126.0983@0.83	127.0983	0.83	4.90E-02	3.75	up	-5.67	down
Lipidomic	148.016@4.77	149.016	4.77	2.73E-03	-9.27	down	-0.07	down
Lipidomic	206.1373@0.80	207.1373	0.8	3.58E-02	3.05	up	-6.95	down
Lipidomic	343.2175@0.80	344.2175	0.8	5.40E-03	-5.5	down	7.19	up
Lipidomic	395.3869@5.27	396.3869	5.27	5.01E-04	-14.47	down	-4.05	down
Lipidomic	440.78@2.04	441.78	2.04	4.59E-02	-6.16	down	-6.14	down
Lipidomic	488.3866@8.15	489.3866	8.15	2.42E-02	11.15	up	5.6	up
Lipidomic	523.4444@3.98	524.4444	3.98	9.90E-03	-7.5	down	-11.5	down
Lipidomic	527.5512@9.70	528.5512	9.7	2.92E-02	-5.36	down	1.47	up
Lipidomic	620.3254@0.87	621.3254	0.87	8.73E-03	-6.95	down	-11	down
Lipidomic	655.7951@3.25	656.7951	3.25	2.77E-02	-9.49	down	-9.6	down
Lipidomic	686.1934@8.07	687.1934	8.07	4.53E-03	10.26	up	1.41	up
Lipidomic	701.2069@5.11	702.2069	5.11	1.23E-02	5.31	up	-5.26	down
Lipidomic	716.2407@3.26	717.2407	3.26	5.48E-03	-10.16	down	-1.61	down
Lipidomic	723.5178@0.86	724.5178	0.86	6.75E-03	-3.71	down	-11.21	down
Lipidomic	724.4988@0.86	725.4988	0.86	1.42E-02	6.15	up	10.45	up
Lipidomic	766.3056@0.80	767.3056	0.8	2.26E-02	9.72	up	2.3	up
Lipidomic	909.7228@9.17	910.7228	9.17	4.15E-02	-0.35	down	-0.71	down
Lipidomic	928.2308@6.76	929.2308	6.76	3.19E-03	8.91	up	9.6	up
Lipidomic	980.2714@9.21	981.2714	9.21	3.22E-02	-5.9	down	1.7	up
Lipidomic	1039.331@8.24	1040.331	8.24	1.50E-02	-4.85	down	4.83	up
Lipidomic	1086.2039@3.25	1087.2039	3.25	1.91E-02	-8.38	down	-7.02	down
Lipidomic	1141.9706@7.54	1142.9706	7.54	2.17E-02	7.71	up	6.04	up
Lipidomic	1218.3806@9.78	1219.3806	9.78	1.61E-02	0.87	up	-8.06	down
Lipidomic	1245.352@9.68	1246.352	9.68	4.80E-02	-2.51	down	-8.79	down
Lipidomic	1303.4269@9.60	1304.4269	9.6	2.46E-02	5.45	up	-0.07	down
Lipidomic	1428.3749@10.01	1429.3749	10.01	2.06E-02	-7.68	down	-0.11	down
Lipidomic	1483.446@9.31	1484.446	9.31	4.89E-02	-7.31	down	1.63	up
Lipidomic	1612.456@10.18	1613.456	10.18	7.74E-03	6.89	up	-3.63	down
Lipidomic	1686.4612@10.25	1687.4612	10.25	1.20E-02	-10.82	down	-3.36	down
Metabolomic	465.3105@8.93	466.3105	8.93	1.76E-04	2.89	up	2.85	up
Metabolomic	736.1996@11.50	737.1996	11.5	1.37E-03	1.24	up	3.87	up
Metabolomic	1203.8696@13.27	1204.8696	13.27	1.53E-03	3.36	up	2.4	up
Metabolomic	301.291@8.56	302.291	8.56	1.78E-03	1.53	up	-3	down
Metabolomic	401.6842@7.33	402.6842	7.33	2.07E-03	3.16	up	0.63	up
Metabolomic	803.3805@7.33	804.3805	7.33	2.70E-03	-3.52	down	-1.45	down
Metabolomic	494.4399@12.93	495.4399	12.93	2.81E-03	2.36	up	2.32	up
Metabolomic	660.4925@7.34	661.4925	7.34	2.98E-03	-1.94	down	0.42	up
Metabolomic	693.0938@5.89	694.0938	5.89	3.04E-03	3.03	up	1.14	up

Metabolomic	541.0597@0.58	542.0597	0.58	3.60E-03	-0.02	down	-2.36	down
Metabolomic	257.2745@9.11	258.2745	9.11	4.61E-03	0.01	up	2.69	up
Metabolomic	276.1407@7.97	277.1407	7.97	4.66E-03	0.03	up	2.39	up
Metabolomic	809.6256@13.48	810.6256	13.48	5.36E-03	2.53	up	-2.31	down
Metabolomic	789.0799@13.34	790.0799	13.34	8.78E-03	1.79	up	2.9	up
Metabolomic	751.6019@13.20	752.6019	13.2	8.82E-03	-0.81	down	3.08	up
Metabolomic	956.6196@11.05	957.6196	11.05	9.62E-03	3.02	up	0.89	up
Metabolomic	892.8324@14.91	893.8324	14.91	1.03E-02	2.1	up	2.69	up
Metabolomic	256.1574@7.17	257.1574	7.17	1.16E-02	-1.23	down	-3.04	down
Metabolomic	555.8421@11.12	556.8421	11.12	1.18E-02	1.19	up	3.13	up
Metabolomic	745.5965@13.32	746.5965	13.32	1.18E-02	3.08	up	2.71	up
Metabolomic	642.4017@8.26	643.4017	8.26	1.20E-02	2.5	up	1.64	up
Metabolomic	809.6336@13.49	810.6336	13.49	1.30E-02	-1.66	down	2.47	up
Metabolomic	866.807@14.85	867.807	14.85	1.34E-02	2.77	up	2.62	up
Metabolomic	754.4539@11.13	755.4539	11.13	1.37E-02	1.18	up	2.87	up
Metabolomic	366.2129@7.33	367.2129	7.33	1.43E-02	-0.11	down	0	down
Metabolomic	171.1246@10.59	172.1246	10.59	1.46E-02	1.92	up	3.29	up
Metabolomic	687.102@5.90	688.102	5.9	1.64E-02	2.02	up	0	down
Metabolomic	612.1499@0.46	613.1499	0.46	1.69E-02	-2.46	down	-0.51	down
Metabolomic	512.2719@8.22	513.2719	8.22	1.72E-02	3.03	up	0.56	up
Metabolomic	985.6554@11.09	986.6554	11.09	1.82E-02	-3.45	down	-2.84	down
Metabolomic	398.2466@10.04	399.2466	10.04	1.83E-02	2.62	up	0.48	up
Metabolomic	368.2334@6.89	369.2334	6.89	1.92E-02	-2.37	down	-0.51	down
Metabolomic	122.0605@7.44	123.0605	7.44	2.00E-02	-2.65	down	0.04	up
Metabolomic	689.5674@12.89	690.5674	12.89	2.00E-02	-3.17	down	-1.58	down
Metabolomic	747.5708@12.97	748.5708	12.97	2.10E-02	-2.15	down	0.63	up
Metabolomic	777.0916@10.56	778.0916	10.56	2.15E-02	0.55	up	-1.75	down
Metabolomic	257.0986@0.48	258.0986	0.48	2.24E-02	-2.27	down	0.69	up
Metabolomic	256.105@1.00	257.105	1	2.27E-02	-1.79	down	0.55	up
Metabolomic	535.2048@12.72	536.2048	12.72	2.36E-02	-3.01	down	0.09	up
Metabolomic	362.2511@11.47	363.2511	11.47	2.36E-02	2.35	up	0.05	up
Metabolomic	426.3541@13.89	427.3541	13.89	2.42E-02	-0.07	down	1.98	up
Metabolomic	814.0889@13.30	815.0889	13.3	2.52E-02	1.67	up	2.21	up
Metabolomic	629.6276@13.51	630.6276	13.51	2.69E-02	2.98	up	2.24	up
Metabolomic	234.1639@9.19	235.1639	9.19	2.78E-02	0	up	2.36	up
Metabolomic	710.465@7.33	711.465	7.33	2.84E-02	-2.79	down	-1.3	down
Metabolomic	268.0814@0.67	269.0814	0.67	2.91E-02	0.01	up	-0.12	down
Metabolomic	733.5959@13.47	734.5959	13.47	2.91E-02	3.39	up	-0.56	down
Metabolomic	705.4679@7.33	706.4679	7.33	2.96E-02	-0.74	down	1.46	up
Metabolomic	976.9137@11.49	977.9137	11.49	3.01E-02	2.99	up	2.26	up
Metabolomic	714.0951@13.01	715.0951	13.01	3.05E-02	2.61	up	0.81	up
Metabolomic	324.9894@0.81	325.9894	0.81	3.08E-02	-0.04	down	2.25	up
Metabolomic	649.4105@11.02	650.4105	11.02	3.12E-02	-1.27	down	-3.1	down
Metabolomic	827.7032@13.82	828.7032	13.82	3.13E-02	-1.15	down	-2.79	down
Metabolomic	590.4992@13.34	591.4992	13.34	3.18E-02	-0.05	down	-2.59	down
Metabolomic	909.5038@14.08	910.5038	14.08	3.19E-02	2.53	up	-0.44	down
Metabolomic	614.5288@13.21	615.5288	13.21	3.32E-02	1.35	up	3.06	up
Metabolomic	611.7945@11.49	612.7945	11.49	3.44E-02	2.25	up	2.41	up
Metabolomic	494.4621@12.92	495.4621	12.92	3.71E-02	-2.33	down	0.19	up
Metabolomic	936.4213@11.50	937.4213	11.5	3.75E-02	2.29	up	2.58	up
Metabolomic	464.8149@11.01	465.8149	11.01	3.89E-02	3.4	up	2.01	up
Metabolomic	1064.4882@10.28	1065.4882	10.28	4.28E-02	-1.63	down	-2.52	down
Metabolomic	911.2679@13.69	912.2679	13.69	4.35E-02	-1.18	down	1.03	up

Metabolomic	666.4007@11.04	667.4007	11.04	4.47E-02	1.19	up	2.32	up
Metabolomic	292.9928@0.50	293.9928	0.5	4.50E-02	1.15	up	2.22	up
Metabolomic	757.6093@13.36	758.6093	13.36	4.58E-02	-0.83	down	2.53	up
Metabolomic	779.0876@10.51	780.0876	10.51	4.62E-02	1.64	up	2.22	up
Metabolomic	751.5926@13.20	752.5926	13.2	4.74E-02	1.59	up	-2.02	down
Metabolomic	843.7998@14.28	844.7998	14.28	4.78E-02	1.82	up	2.75	up
Metabolomic	704.1738@10.44	705.1738	10.44	4.78E-02	-1.16	down	-2.3	down
Metabolomic	718.4498@12.25	719.4498	12.25	4.84E-02	0.62	up	2.68	up
Metabolomic	605.3785@11.58	606.3785	11.58	4.86E-02	1.22	up	-1.37	down

Table S3. Class representative and extraction internal standards added to the samples in untargeted metabolomic and lipidomics analysis.

Compound	Source	Identifier
L-Phenylalanine-1-13C	Sigma	490091
1,3(d5)-dihexadecanoyl-glycerol	Avanti Polar Lipids	110537
1,3(d5)-dihexadecanoyl-2-octadecanoyl-glycerol	Avanti Polar Lipids	110543
1-hexadecanoyl(d31)-2-(9Z-octadecenoyl)-sn-glycero-3-phosphate	Avanti Polar Lipids	110920
1-hexadecanoyl(d31)-2-(9Z-octadecenoyl)-sn-glycero-3-phosphocholine	Avanti Polar Lipids	110918
1-hexadecanoyl(d31)-2-(9Z-octadecenoyl)-sn-glycero-3-phosphoethanolamine	Avanti Polar Lipids	110921
1-hexadecanoyl-2-(9Z-octadecenoyl)-sn-glycero-3-phospho-(1'-rac-glycerol-1',1',2',3',3'-d5)	Avanti Polar Lipids	110899
1-hexadecanoyl(d31)-2-(9Z-octadecenoyl)-sn-glycero-3-phospho-myo-inositol	Avanti Polar Lipids	110923
1-hexadecanoyl(d31)-2-(9Z-octadecenoyl)-sn-glycero-3-[phospho-L-serine]	Avanti Polar Lipids	110922
26:0-d4 Lyso PC	Avanti Polar Lipids	860389
18:1 Chol (D7) ester	Avanti Polar Lipids	111015
cholest-5-en-3ß-ol (d7)	Avanti Polar Lipids	LM-4100
D-erythro-sphingosine-d7	Avanti Polar Lipids	860657
D-erythro-sphingosine-d7-1-phosphate	Avanti Polar Lipids	860659
N-palmitoyl-d31-D-erythro-sphingosine	Avanti Polar Lipids	868516
N-palmitoyl-d31-D-erythro-sphingosylphosphorylcholine	Avanti Polar Lipids	868584
Octadecanoic acid-2,2-d2	Sigma Aldrich	19905-58-9
